# Hydrogel Properties and Their Impact on Regenerative Medicine and Tissue Engineering

**DOI:** 10.3390/molecules25245795

**Published:** 2020-12-08

**Authors:** Adam Chyzy, Marta E. Plonska-Brzezinska

**Affiliations:** Department of Organic Chemistry, Faculty of Pharmacy with the Division of Laboratory Medicine, Medical University of Bialystok, Mickiewicza 2A, 15-222 Bialystok, Poland; adam.chyzy@umb.edu.pl

**Keywords:** hydrogel, physicochemical properties, regenerative medicine, tissue engineering

## Abstract

Hydrogels (HGs), as three-dimensional structures, are widely used in modern medicine, including regenerative medicine. The use of HGs in wound treatment and tissue engineering is a rapidly developing sector of medicine. The unique properties of HGs allow researchers to easily modify them to maximize their potential. Herein, we describe the physicochemical properties of HGs, which determine their subsequent applications in regenerative medicine and tissue engineering. Examples of chemical modifications of HGs and their applications are described based on the latest scientific reports.

## 1. Introduction

Hydrogels (HGs) are three-dimensional (3D) structures of relatively uncomplicated design. They are mainly composed of long chains of polymers, forming a complex matrix in which the spaces between polymeric chains are filled with water molecules. Depending on the chemical nature of the polymer and the degree of its crosslinking, the properties of HG matrices differ widely. HGs are used extensively in medicine and pharmaceutical sciences. They are used (in addition to the later presented applications in regenerative medicine and tissue engineering) mainly as drug carriers [1,2,3], in wound dressings [4,5,6], in contact lenses [7,8,9] or in gene therapy [10,11,12].

Tissue engineering is focused on producing a substitute for a specific tissue or an entire organ. The main application of this process is in cell cultures, which are placed on appropriately constructed scaffolds for growing and subsequent transplantation into the organism. Cell cultures utilized explicitly in this field of science are mainly stem cells due to their pluri- or multipotency and unlimited expansion. A broader concept that includes tissue engineering is regenerative medicine, whose objective is to stimulate self-regenerative mechanisms of the human body. This process can be implemented using stem cells or growth factors. If the organism is not capable of regenerating itself, regenerative medicine exploits the benefits of tissue engineering. This approach enables the transplantation of tissues or entire organs that have been grown in laboratory conditions (e.g., from cells obtained from a patient), so the rejection risk is minimized.

## 2. Physicochemical Properties

This section focuses on the physical and chemical properties that define HGs as spatial structures. These structural properties are largely responsible for the behavior of HGs and, consequently, for their final application. The physicochemical properties of HGs have a particularly large impact on the final properties of the materials and their biological suitability. Below are some parameters/properties of the materials that should be taken into account in material engineering due to their implications for medical applications.

### 2.1. Swelling Ratio

Swelling is a physical process in which the material increases its volume, and consequently, its mass, by absorbing liquid. As the size of the studied materials increases, the original shape is preserved. Swelling is one of the fundamental properties characteristic of most HGs. Depending on the polymer matrix used to create the gel, it can absorb many times more water than its dry mass [13]. It is assumed that increasing the HG’s mass (e.g., more water can be absorbed) increases its sorption capacity. The swelling process is strongly affected by the presence of hydrophilic groups in the gel structure [14]. Hydroxyl and carboxylic groups are the most desirable in this regard. In the presence of water, they are the first to hydrate, causing the solvent molecules to penetrate the gel matrix and remain there until the equilibrium state is reached. Hydrogen bonds formed between water molecules and the functional groups of the polymeric chain stabilize the HG structure.

The basic value and unit characteristic of the described process is the swelling ratio (SW). The SW is defined as the increase in HG weight due to water absorption [15] and is expressed by Equation (1)

(1) $$\mathrm{SW}\;=\;\frac{m_{water}}{m_{dried}}\;=\;\frac{m_{swollen}{\;-\;m}_{dried}}{m_{dried}}$$

where *m_water_* is the amount of water absorbed by the gel, *m_dried_* is the mass of the polymer matrix before swelling, and *m_swollen_* is the mass of the polymer gel after swelling in equilibrium.

The most popular theoretical model of the swelling process, ‘equilibrium state’, was proposed in 1943 by Flory and Rehner [16], and in 2020, it was updated by Peppas et al. [17] and Richbourg et al. with additional variables [18]. This model assumes that the maximally swollen gel is in thermodynamic equilibrium with the solution in which it is immersed. Originally, this model was described by Equation (2)

(2) $$\Delta G_{\mathrm{total}}\;=\;\Delta G_{elastic\;}+\;\Delta G_{mixing}$$

where ∆*G_total_* is the total free energy change, ∆*G_elastic_* is the value of polymer chain retraction forces, and ∆*G_mixing_* refers to the spontaneous mixing of solvent molecules in the gel structure at equilibrium. Considering the number of solvent molecules, assuming constant values of temperature (*T*) and pressure (*p*), Equation (2) can be transformed into Equation (3), which is based on the chemical potential

(3) $$\frac{\partial (\Delta G_{total})}{\partial n_{1}}\;=\;\frac{\partial (\Delta G_{elastic})}{\partial n_{1}}\;+\;\frac{\partial (\Delta G_{mixing})}{\partial n_{1}}\;=\;\Delta \mu_{total\;}\;=\;\Delta \mu_{elastic}\;+\;\Delta \mu_{mixing}$$

Here, the *n*_1_ value refers to the number of molecules in the solution and the ∆*µ* values to chemical potentials. Briefly, if the total chemical potential is zero, the same number of molecules penetrate as those that exit the HG network.

The responses mentioned above assume that there are no charged particles (ions) in the HG. Their presence inside and outside the matrix requires more complex formulas and calculations. Apart from the free energy of ∆*G_elastic_* and ∆*G_mixing_*, the ionic free energy (∆*G_ionic_*) should be added to the general equation (Equation (2))

(4) $$\Delta G_{total}\;=\;\Delta G_{elastic\;}+\;\Delta G_{mixing}\;+\;\Delta G_{ionic}$$

After differentiation (assuming constant *T* and *p* values), ∆*G_total_* becomes the ∆*µ_ionic_* value and includes concentrations of individual (positively and negatively charged) ions inside and outside the HG structure. Equation (4) can thus be described as

(5) $$\Delta \mu_{total}\;=\;\Delta \mu_{elastic}\;+\;\Delta \mu_{mixing}\;+\;\Delta \mu_{ionic}$$

In modern pharmacy, the swelling process provides the potential to use HGs as carriers of therapeutic substances. In regenerative medicine, especially in infected wound treatment, with the use of these structures, it is possible to deliver antibacterial substances, including antibiotics [19,20,21] and silver particles [22,23,24]. These structures can also be used in tissue engineering as a promising scaffold for cell or protein delivery systems [25].

Placing the non-swollen gel in a drug solution can allow both solvent and drug molecules to penetrate the polymer network. This process is conditioned by diffusion rules according to Fick’s laws. Solvent molecules cause the expansion and swelling of the gel network, while drug molecules cause its ‘loading’.

Kim et al. described the amount of drug absorbed in the polymeric matrix using Equations (6) and (7) [26]. However, these equations assume that the gel and solution are in equilibrium.

The lower limit (for gel in the swollen state)

(6) $$\frac{V_{s}}{W_{p}}{\;*\;C}_{o}$$

The upper limit (for gel in the swollen and the polymer state)

(7) $$\frac{{(V}_{s}{\;+\;\mathrm{KV}}_{p})}{W_{p}}{\;*\;C}_{o}$$

where *V_s_* and *V_p_* are the volumes of absorbed solvent and dried polymer, respectively. *W_p_* is the weight of the dried polymer, K is the value of the partition coefficient between the drug solution and polymer network, and *C_o_* is the drug concentration in solution.

### 2.2. Porosity

Porosity (*p_por_*) is the ratio of the void volume (*V_por_*) in the tested material to its total volume (*V*). In general, the porosity of the material can be expressed by Equation (8)

(8) $$p_{por}=\frac{V_{por}}{V}$$

**Figure 1:**
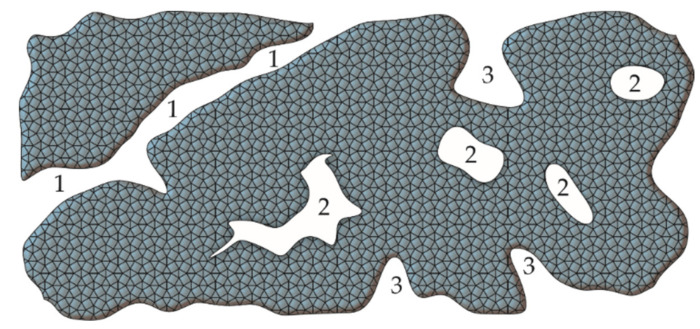
Types of pores in the material: (**1**) transport pores, (**2**) closed pores, (**3**) open pores.

It is widely accepted that pores can be divided into open and closed pores (Figure 1). The former have a connection with the outer space, and the latter do not.

In addition to the mere presence of pores in the structure of the material, their size is also crucial. According to the International Union of Pure and Applied Chemistry classification, the diameter of pores can be distinguished as follows: (i) micropores with a diameter below 2 nm; (ii) mesopores with diameters between 2 and 50 nm; and (iii) macropores with diameters above 50 nm. It should be noted, however, that from a biological and medical point of view, pores with a larger diameter and their accessibility to biological fluids are important (Figure 1). In this regard, a different division of the pores has been proposed, taking into account their diameter and applications. Elbert et al., considering the differentiation of gel structures, propose the following classification [27]: (i) nanopores with diameters below 100 nm; (ii) micropores with diameters between 100 nm and 1 µm; and (iii) macropores with diameters above 1 µm. The pore size, in terms of the HG’s use, can be differentiated as follows [28,29]:

- c.a. 5 µm for neovascularisation;
- c.a. 20 µm for hepatocyte ingrowth;
- between 5 and 15 µm for fibroblast ingrowth;
- between 20 and 125 µm for adult mammalian cells;
- between 40 and 100 µm for osteoid ingrowth;
- between 100 and 350 µm for bone tissue regeneration;
- above 500 µm for fibrovascular tissue development.

To prepare a porous structure of the materials [30,31,32], several methods are used, such as porogen templating [33], gas foaming [34], bicontinuous emulsion templating [35], cryogelation [36], 3D printing [37], electrospinning [38], freeze-drying [39], and inverse opal hydrogelation [40].

Porogen templating is the most commonly used method. It involves the use of special substances, porogens, to forming a porous structure in a material. This process occurs as follows: the porogen takes up part of the HG space during gelation and matrix formation, after which it is removed (e.g., by washing). As a result, the porous material is obtained. The selection of a suitable substance that can be used as a porogen is constrained by several requirements, including an inert character, ability to mix with the reaction solution, and high boiling point. The porogen cannot react or polymerize with the monomer, but must be compatible with the chosen method of reaction initiation [41]. Various substances are used as porogens. The most popular is sodium chloride due to its availability and low cost. Tran et al. designed a new method that has achieved greater control of the pore size in the material [42]. In addition to NaCl, mannitol [33], Na_2_SO_4_ [43], supercritical carbon dioxide [44] and many others have been used for the formation of porous materials [41]. Other methods mentioned above are summarized in Table 1.

The size of the pores in the material is essential for its further use. Therefore, it is crucial to control this parameter at the early stage of creating an HG structure. The appropriate gelation process, selection of the best method to obtain a porous structure and choice of porogen have an impact on the simulation of in vivo conditions, especially in tissue engineering and regenerative medicine [32,45].

### 2.3. Rheology

HGs are qualified as semisolid forms of drug carriers in pharmaceutical sciences. Moreover, like any semisolid body, their properties can be described by rheological parameters. Rheology is a branch of continuum mechanics that deals with plastic deformations and the flow of materials. It is based on the three Reiner’s axioms [46]:

1. All materials exposed to isotropic stress behave like a perfectly elastic material.
2. Each material has all rheological properties, although to different degrees.
3. The rheological equation of a simpler object can be derived from that of a more complex object by comparing the relevant parameters to zero.

The relevant parameters are shear stress, shear rate, viscosity and thixotropy. Shear stress (*τ*) is the ratio of force (*F*) to area (*A*), which is described by Equation (9)

(9) $$\tau =\frac{F}{A}$$

The effect of *τ* on the material is deformation (plastic, elastic or flow). Deformation (γ) is the change in the material (i.e., the change in mutual position of the elements of the body), which, under *F*, causes a displacement of the element by a distance (*l*) (*y* is the distance between boundary layers). This ratio is described by the formula

(10) $$\gamma =\frac{dl}{dy}$$

The shear rate ($\dot{\gamma }$) describes the speed at which an object deforms as a result of *τ*. The unit of shear rate is s^−1^, and the formula describing this parameter is represented by Equation (11), where *v* is the speed gradient and *t* is the time at which the deformation occurs

(11) $$\dot{\gamma }=\frac{dv}{dy}=\frac{d\gamma }{dt}$$

Knowing the above, it is possible to determine the viscosity of the material. Assuming the internal laminar system of the tested object, the dynamic viscosity (*η*) is defined as the resistance that arises when these layers move against each other. *η* is expressed as the ratio of *τ* to $\dot{\gamma }$ (Equation (12))

(12) $$\eta =\frac{\tau }{\dot{\gamma }}$$

The unit of dynamic viscosity is Pa·s. The *η* value is highly dependent on two factors: *T* and *p* (pressure). Increasing the *T* value causes a decrease in *η*. In contrast, increasing *p* causes an increase in system *η.*

Thixotropy is an important characteristic of most non-Newtonian fluids, including HGs. It is described as a temporary decrease in the viscosity of material under the influence of shear force and (after a certain time) its return to initial viscosity values. This behavior is explained by the possibility of the gel–sol transition or the destruction of the internal gel structure under the influence of an external force, followed by its slow and gradual restoration. Understanding rheological parameters is essential in planning the use of a specific HG, especially in tissue engineering. Selection of appropriate values of viscosity and shear stress allows the application of an HG with a syringe [47,48], painting with a brush directly on the tissue [49] or squeezing it from a tube to be applied on the skin.

### 2.4. Biocompatibility

Many factors must be considered when developing new material for medical use, especially for internal administration. One of the most crucial properties of materials for biological and medical uses is their biocompatibility. Over the years, several definitions of biocompatibility have been used. It can be defined most simply as the ability of medical material to exist in the body without causing adverse effects. It is assumed that this material will not engage the immune system, will not have an adverse impact on the surrounding tissues or cells and will therefore be inert per se. In principle, most HGs, due to their structure, consistency and high water content, show high biocompatibility with living tissues [50]. To increase the biocompatibility of the HG structure, it is essential to eliminate any unreacted amounts of substrates, monomers, initiators, and other substances used in the HG preparation process that may themselves cause adverse effects in the body.

The main reason why biocompatible HGs are created is their use in regenerative medicine and tissue engineering. They are primarily used to treat wounds [51,52], administer drugs parenterally [53,54], and imitate the environment for cell growth and development [55,56]. An important issue that is closely related to biocompatible materials, including HGs, is the concept of biomimetics. Biomimetics (also called bionics or biomimicry) is generally referred to as the design and creation of objects based on the pattern of living organisms. Pharmaceutically, biomimetics is therefore concerned with the development of a drug carrier material that most accurately imitates part of the human body. It is essential to know all the processes occurring in the human body, especially immune reactions, which are the main factors governing the potential use of any pharmaceutical material (e.g., a drug carrier).

In terms of HGs, biomimetics involves the design of the HG structure in such way that the human body’s immune system does not treat it as a foreign object while facilitating the delivery of the drug to the target site. This outcome is achieved through the selection of the appropriate matrix-forming polymer as well as the addition of substances imitating the tissue’s environment. These compounds may be growth factors, peptides, or proteins [57].

**Figure 2:**
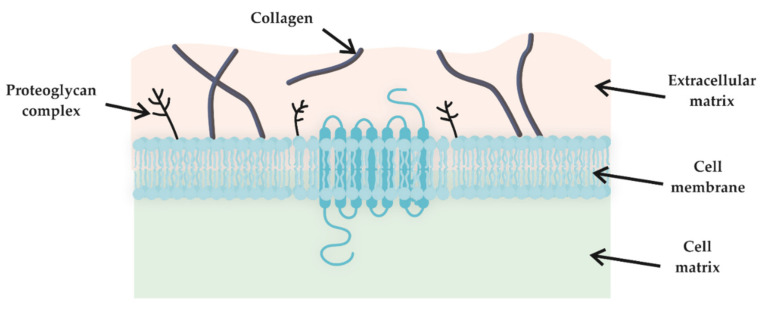
Extracellular matrix localization in living tissue.

In tissue engineering and regenerative medicine, one of the most important applications for which biocompatible HGs are used, in addition to wound treatment, is the mimicking of the extracellular matrix (ECM). This matrix is produced by cells and provides a living habitat that fills the space between cells (Figure 2). The ECM is composed mainly of collagen, glycosaminoglycans (such as hyaluronic acid) and growth factors [58]. Due to their structural similarity and the possibility of functional modification, HGs are commonly used as artificial ECM. Additionally, HGs can support cell division, attachment, and molecular response [59].

Before a material can be classified as biocompatible, it must be proven that neither the material per se nor its degradation products are toxic to the body. To confirm this, several tests are carried out, which Porto has divided into three levels: primary, secondary, and preclinical [60]. The first two are all in vitro and in vivo tests, while the preclinical level involves the administration of the material to humans. The in vitro test assumes the determination of the cytotoxicity of the material using different cell lines, including human and animal cell lines. Keratinocytes, lymphocytes, fibroblasts, and macrophages can be used. In vivo tests involve administering the material subcutaneously, intramuscularly, or epidermally to the model animal and describing the positive or negative effects. The three levels of tests allow researchers to determine the impact the material will have on the human body under clinical conditions. A material that passes all three tests can be approved for commercial use.

Biodegradation is the process in which the structure of a single material is degraded to simpler products under the influence of biological activity. Along with biocompatibility, biodegradation is another crucial feature of medical material. In some cases, despite the positive results of the cytotoxicity tests for the materials, the products of their decomposition could be toxic for the human organism, precluding the use of these structures in further research. For example, a structure that is intended to release an active substance over a long period must not degrade too quickly and violently. On the other hand, it should not remain in the body for too long, necessitating surgical removal (such as nondegradable implants).

There are four major mechanisms responsible for the decomposition of biodegradable polymers, including HGs: (i) hydrolysis, (ii) oxidation, (iii) enzymatic decomposition and (iv) physical processes. Hydrolytic degradation assumes in the presence of water in the tissue, more sensitive bonds in the polymer chain start to degrade, which shortens the polymer chains.

Oxidative degradation is caused by activation of the body’s immune system. The presence of a foreign object in the body causes the involvement of appropriate immune cells (e.g., macrophages, neutrophils), which begin to produce peroxides as a defense response. Peroxides, through a series of chemical reactions, lead to the rupture of the polymer chain and its degradation. Enzymatic decomposition is based on the participation of specific enzymes present in tissues. It is strictly dependent on individual diversity and on the tissue the material is applied to. Physical decomposition, as opposed to chemical processes, relates to frictional forces and movement of the whole organism as well as many other physical factors that mechanically cause decomposition of an implanted material.

### 2.5. Self-Healing

Self-healing of HGs is an interesting phenomenon, inspired by the processes that occur in nature. It is defined as the HG’s response to a harmful factor by rebuilding its structure and returning to its original state without the intervention of external factors [61]. This process seems to be extremely beneficial and useful in terms of use in regenerative medicine and tissue engineering, especially when considering HGs for internal parenteral administration. Self-healing may provide an increased duration of HG in the body and thus prolong therapy and improve patient treatment. However, HGs may be considered self-healing materials that fulfil several requirements [61]. First, the HG should be made of nontoxic and economically beneficial material using uncomplicated methods of preparation. An HG should not degrade rapidly after application to tissues. The self-healing process itself should initiate after the occurrence of damage and should be repeatable and independent of external factors. HGs should have adequate rheological and mechanical properties selected for application, and after the self-healing process, these properties should not differ from those before the damage factor. However, these are only guidelines for an ideal state, and it is often difficult to fulfil all these requirements simultaneously. Considering the aforementioned phenomenon of thixotropy, it may be recognized as self-healing. However, there is a small difference between them. Self-healing only occurs when the harmful factor stops working, while thixotropic effects occur when the harmful factor is active and after it has ceased [62].

**Figure 3:**
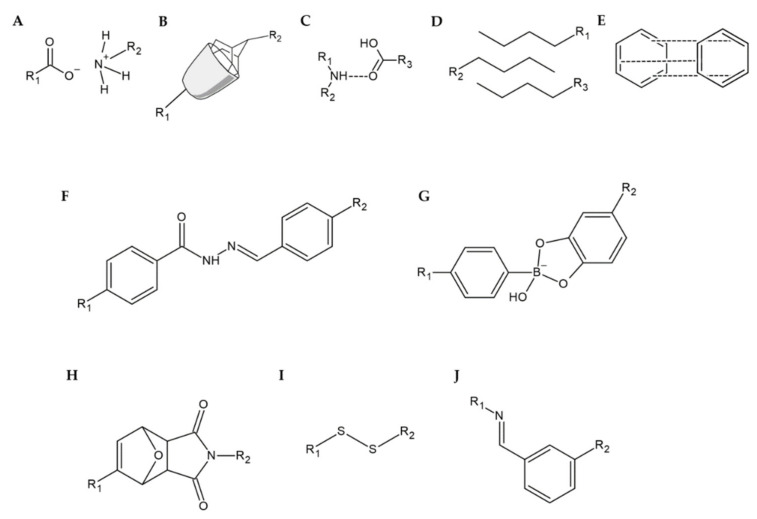
Example of self-healing mechanisms supported by (**A**) electrostatic interactions, (**B**) host–guest interactions, (**C**) hydrogen bonds, (**D**) hydrophobic interactions, (**E**) π–π stacking, (**F**) acylhydrazone bonds, (**G**) boronate-diol complexation, (**H**) Diels–Alder reaction, (**I**) disulfide bonds, and (**J**) imine bonds.

Several factors may be considered when specifying the physical or chemical properties that affect self-healing. Wang et al. divide these mechanisms into physical and chemical [63]; Tu et al. specify this classification, calling physical mechanisms noncovalent and chemical mechanisms covalent [64]; while Fan et al. look at the classification much more comprehensively and divide the mechanisms into intrinsic and extrinsic [65]. The classification of self-healing mechanisms is summarized in Table 2 and is presented in Figure 3.

Electrostatic (or ionic) interaction is based on the interaction between ions incorporated into the HG matrix (Figure 3A). By the mutual attraction of oppositely charged ions or ions and polymer chains, the HG structure can be reconstructed, e.g., between two pieces of HG placed on each other [66]. The electrostatic attraction forces between polyethylene glycol (PEG) and chitosan or between PEG and alginate formed an HG that had recovered entirely from damage, which was confirmed with almost identical stress test results. The PEG/alginate HG also returned to its original condition, but its strength was lower (but still high) compared to its original form. Pu et al. created a self-healing HG based on electrostatic effects between the different moieties of the HG [67]. The difference from the results previously described was that the HG structure (polyhedral oligomeric silsesquioxane-based matrix) healed much faster.

The mechanism that engages two or more chemical compounds, causing them to aggregate, is host–guest interaction (Figure 3B). In this process, the functional group of one compound, called the ‘guest’, is enclosed within the chemical group of the other compound, called the ‘host’. The most widely used hosts are cyclodextrins, crown ethers, calixarenes, pillararenes, and cucurbiturils [65]. Zhu et al. presented a thermosensitive HG consisting of poly(*N*-isopropylcrylamide) (PNIPAM), adamantyl as guest and cyclodextrin as host [72]. The HG healed quickly, and the process was repetitive. In addition, expectations are high that this HG can be used to treat wounds under mild temperature control. Liu et al. created an HG containing cyclodextrin (host) and amino acids (guest) [73]. Again, no significant differences between the initial and healed HG were observed. Another study presented the host-guest relationship between cyclodextrin and azobenzene, showing that the additional presence of agarose improved and enhanced the self-healing properties of the HG structure [74].

A hydrogen bond is an interaction between a hydrogen atom in one molecule and an acceptor in another molecule with an unpaired electron (Figure 3C). The hydrogen atom that is involved in this process must be derived from the hydrogen-high electronegative atom pair, which is usually represented by nitrogen, oxygen, or fluorine atoms [77]. An example of an HG in which this interaction is observed is the structure developed by Sharma et al. [78] containing chitosan, which is a donor of hydrogen, and poly-acryloyl-phenylalanine, which is a hydrogen acceptor. No differences were observed between the HG structures before and after healing. Scientists believe that this material can be used as a self-healing dressing HG or as a drug carrier. Additionally, researchers led by Song R. presented the formation of an HG containing cordycepin and chitosan [79]. The experiments showed that the HG’s structure was stabilized by hydrogen bonds originating from chitosan. Additionally, the HG showed antibacterial properties in vitro, allowed faster epithelization of skin damage and had an impact on increasing the expression of epithelial regeneration markers, making it perfectly suited for regenerative medicine.

Hydrophobic interactions in self-healing are based on the addition of hydrophobic chains or groups to the polymer matrix, which forms a larger 3D structure of HG (Figure 3D). According to the self-organization of the component, when exposed to water, aggregates are formed. Thus, the HG structure is rebuilt after the harmful factor is applied. This concept was adopted by Zhao et al. to create an HG containing poly(ethylene glycol)-*b*-polypeptide. The HG was able to enclose and administer doxorubicin, a drug with hydrophobic properties [83]. Voorhaar et al. prepared an HG with an ABA block structure, where the hydrophilic moieties were in the center (B) and the hydrophobic moieties (A) responsible for structure crosslinking were in the terminal end [84].

π-π stacking is based on the mutual interaction of two or more aromatic systems (Figure 3E). The attraction forces are generated between the electron clouds of the aromatic rings of two moieties. This interaction was observed by Liang et al. [87] between the rings of hyaluronic acid and dopamine. Additionally, the HG developed has antibacterial properties and may be used as a vehicle for drugs, which enables its application in regenerative medicine, especially in wound treatment [88].

Acylhydrazone bonds are an example of the chemical mechanism of the self-healing process occurring in HGs (Figure 3F). These bonds are mainly developed between aldehyde or ketone groups and imine groups derived from acylhydrazine. An example of an HG in which these associations were used is the structure developed by Xiao et al. [68]. The HG exhibited a significant possibility of self-healing, which is explained by the ability of acylhydrazone bonds to recreate these bonds reversibly. Additionally, the authors assumed that due to the low cytotoxicity and biocompatibility of the gel structure, the material might be used for tissue engineering and regenerative medicine in the future. Similarly, Lü et al. presented an injectable HG, formed in vivo, whose healing process was also based on acylhydrazone bonds [69]. Interestingly, due to the inclusion in the matrix of bone morphogenetic protein 4, which is a bone growth factor, the HG structure can be used for the regeneration of injured bones.

Boronate-diol complexation involves the formation of ester bonds between boronic acid and hydroxyl groups of another molecule (Figure 3G). Therefore, this type of self-healing mechanism is also called a boronate ester bond. Shi et al. developed an HG whose structure contained phenylboronic acid, hyaluronic acid, and polyvinyl alcohol [75]. They demonstrated that the self-healing mechanism for this material was based on a boronate-diol complexation mechanism. Moreover, the HG structure was reactive oxygen species dependent, which implies the possibility of using this HG to deliver therapeutic substances. In addition, due to the appropriate rheological properties, the material is suitable for injection into the body or for 3D printing used in tissue engineering. Similarly, the polyacrylamide HG modified with the addition of gold nanoparticles and nanorods uses boronate ester bonds for its stabilization [76]. In addition to its ability to self-heal, this HG showed the potential for controlled release of therapeutic substances. Interestingly, the release of the drug occurred mainly when treating the HG structure with UV radiation. Therefore, it was assumed that this HG could be widely used in medicine.

The Diels–Alder reaction is an example of a cycloaddition reaction resulting in a cyclic compound (Figure 3H). This reaction occurs between two substrates: a diene and a dienophile. Banerjee et al. described the synthesis of an HG whose self-healing mechanism is based on the Diels–Alder reaction [80]. After the harmful factor was applied, the HG was exposed to a high temperature of 170 °C for 1 h (5 degrees above Diels–Alder’s retro-reaction point), after which it was cooled down. Due to the high temperature (relative to the human body temperature) at which the Diels–Alder reaction occurs, it is often combined with other self-healing mechanisms. This concept was adopted by two groups of researchers, Li et al. and Ghanian et al., who used the Diels–Alder reaction and the creation of coordination bonds with iron or calcium ions, respectively [81,82].

Disulfide bonds are known for their low requirements for reaction conditions (Figure 3I). The principle of their formation is based on the thiol/disulfide exchange reaction, which is sensitive to changes in oxidative-reduction potential and pH. To create an HG, bovine serum albumin (BSA) was used, which is rich in disulfide bonds [85]. The addition of hydrogen peroxide induced the self-healing process of the HG. This process led to the breaking of disulfide bonds of single albumin, which resulted in the formation of free sulfhydryl groups, which consequently ‘attacked’ another BSA molecule, causing a new disulfide bond. As a result, the HG exhibited self-healing properties very quickly (in two minutes). Researchers assumed that due to these described properties and the possibility of its injection, this material could be used in regenerative medicine and tissue engineering. Song et al. prepared HG containing Pluronic F127 and lipoic acid, which is a source of sulfur [86]. In this case, the self-treatment process was induced by UV radiation on the HG, and during this interaction, disulfide bonds were formed. Additionally, preliminary research has shown that this structure is a promising drug carrier.

The nucleophilic attack reaction between the primary amine and aldehyde group results in the formation of the imine bond (Schiff base) (Figure 3J). As a rule, the aromatic Schiff base is more stable than the aliphatic bases. An interesting application for this kind of bond was shown by Huang et al., who formed an HG containing carboxymethyl chitosan and cellulose nanocrystals [89]. They noticed that the HG was suitable for further use 5 minutes after being injured. The most impressive finding is that this material has an extraordinary ability to dissolve immediately after being exposed to an amino acid solution. According to these results, this phenomenon can be used for painless removal of wound dressings based on this hydrogel. Du et al. synthesized a chitosan-dextran HG with the ability to self-heal and accelerate wound healing [90].

The extrinsic mechanisms are mainly based on the presence of reservoirs with a healing factor in the HG structure. Under the destructive effect of the irritation factor, these reservoirs are destroyed, and the healing agent is released into the HG, causing its repair. These reservoirs can be represented, for example, by microcapsules [70,71].

## 3. Chemical Modification of HGs

### 3.1. pH Responsiveness

One example of smart HGs that respond to the environmental conditions in which they are located is a pH-resistant HG. The principle that guides researchers in the design of this type of material is that in the human body, a wide range of pH values is present depending on the location. Thus, under physiological conditions in the stomach, a pH below 4.0 can be observed; blood and tissues have a pH in the range of 7.35–7.45; and in muscles (not exposed to high physical stress), the pH is 7.0, while the pH of skin varies between 4.0 and 6.0. The pH values of wounds range from neutral to slightly alkaline, which is a significant difference compared to the pH of healthy skin. On the other hand, the pH of tumors is slightly lower than the pH of the blood and ranges between 6.8 and 7.0 [91].

**Figure 4:**
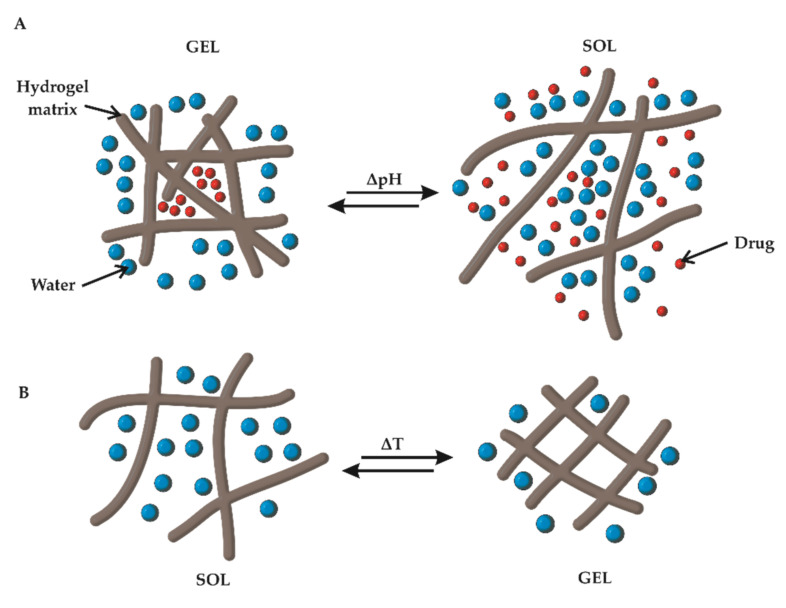
Sol–gel transition of a (**A**) pH-responsive HG and (**B**) thermoresponsive HG.

To obtain material that responds to fluctuations in the pH values, the protonation or deprotonation of the functional groups incorporated in the HG matrix is used (Figure 4A). The effect that is achieved by this process is based on the transition from ‘sol’ to ‘gel’ after its application to the body or due to stimuli caused by pathophysiological or physiological changes in the body. In addition to the crosslinking of the gel, prolongation of the release time of the active substance or drug release caused by pH-changing stimuli was achieved.

Liang et al., while developing a chitosan-based HG, followed the non-physiological pH value of cancer lesions [92]. Due to a slightly decreased pH of the tumor cells, the amine groups incorporated in the HG may be protonated, and thus, they tend to repel each other, causing the HG to swell and increase the pore size of the HG matrix. As a result, in ‘the tumor’s environment’, increasing drug release is observed when compared to healthy tissue with physiological pH. Therefore, this material may be a suitable choice for a local and personalized drug delivery system. A similar principle was used by Pham et al. [93], where an HG was synthesized using three components: gelatine, PEG, and L-Dopa. Scientists have shown that increasing the pH value resulted in a decrease in the swelling ratio of HG, and additionally, a lower pH forced the release of a larger amount of active anticancer substance, which influenced the effectiveness of the therapy.

An alginate-based HG, formed of microparticles, with the addition of CaCO_3_ successfully extended the release period of the active substance [94]. As a result, the model drug was released even after 96 h, when it was tested at pH imitating the wound environment (i.e., mildly acidic, 6.4). When physiological pH (7.4) was used, the whole drug was released after 10 h. This process showed that this HG could be used as a vehicle to deliver drugs (e.g., antibiotics) directly to the wounds, which can significantly enhance healing and regeneration.

The influence of pH on the HG structure was also studied by Zhao et al., who synthesised an HG based on carboxymethyl chitosan and amorphous calcium phosphate [95]. The material was characterized by rheological properties that were dependent on the pH of the surroundings. Thus, at pH similar to the physiological value, the HG solidified, and it was cohesive even up to 36 h. However, when the pH was acidified, the HG’s structure became more liquid but maintained its structure. Interestingly, at physiological pH, the structure of the HG showed properties that allowed them to be continuously injected through a syringe, even after 36 h. These features were used to encapsulate mesenchymal stem cells and to determine in vitro and in vivo osteoinductive properties. It has been shown that the pH-dependent structure could be employed to support bone regeneration processes in regenerative medicine.

### 3.2. Thermoresponsiveness

Thermoresponsive HGs are materials that have a reversible phase transition capability depending on the temperature of their environment. Two important terms must be considered when taking into account this property: lower critical solution temperature (LCST) and upper critical solution temperature (UCST). The LCST is the temperature at which a solution shows a phase transition to gel, i.e., the material becomes less soluble when warmed. On the other hand, UCST is the temperature above which a phase transition from gel to sol occurs, causing the material to become more soluble when heated (Figure 4B). Above the LCST, the collapse of polymer chains is observed, which causes a decrease in the volume and swelling ratio of HGs [96]. Several factors affect the thermodependent properties of HGs. Maeda mentions these factors in reference to polyethylene glycol–poly(lactic-*co*-glycolic acid) (PEG-PLGA) HGs [97]. These factors include the mass ratio of the monomers that build the polymer chain, molecular weight distribution, salt concentration, and type of ions.

Cao et al. described the mechanism of the peptide-PNIPAM HG response to the temperature factor [98]. Their results showed that the polymer chains below the LCST were highly hydrated, and the interaction between them was very weak. Above the LCST (33 °C), the polymeric chains were aggregated with peptide nanofibrils, which resulted in the formation of an entangled, more chaotic gel structure. When the ambient temperature dropped below 33 °C, the gel structure returned to the sol phase, which confirmed the reversible property of the phase transition. Interestingly, scientists have succeeded in encapsulating the antibacterial peptide G(IIKK)_3_I-NH_2_ in an HG matrix, which is highly selective against bacteria and cancer. It has been shown that in laboratory conditions simulating human body temperature, the release of the peptide had a linear dependence on time, which may suggest a constant and steady release in the human body. HG could be successfully used for the administration of active substances as an injection in tissue engineering.

An example of the therapeutic use of thermodependent HG in regenerative medicine was presented by Varma et al. [99]. Methacrylated carboxymethyl cellulose (CMC) and methylcellulose formed an HG that could be utilized as a filling of the intervertebral cavity after removal of the intervertebral disc in degenerative spinal diseases. Thermodependence was used here to allow this material to be injected in liquid form, where under the influence of body temperature, the polymer structure was crosslinked and gelled. Due to its good rheological properties, the size and plasticity of the original polymeric network were successfully restored.

HG containing polyisocyanopeptide was used in tissue engineering [100]. It was proven that the material was suitable for cell differentiation and growth in fibroblasts, endothelial cells, adipose-derived stem cells, and melanoma cells. These findings provide useful evidence for the application of HGs in many areas of tissue engineering. Interestingly, the structure prepared by scientists under in vitro conditions showed the ability to produce complex structures, such as blood capillaries. The ability of an HG to support cell proliferation was also indicated by Zhao et al. [101]. Mesenchymal stem cells were incorporated into the HG matrix, making it possible to achieve osteoinductive and osteoconductive properties.

**Figure 5:**
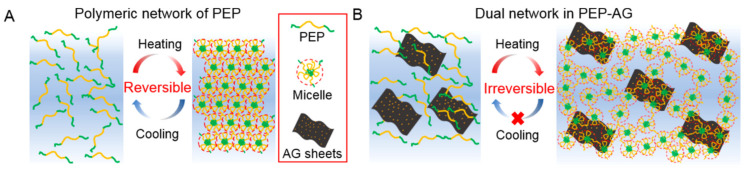
Illustration of the thermoresponsive mechanism of (**A**) PEP and (**B**) PEP-AG HGs. Adapted with permission from [102]. Copyright (2020) American Chemical Society.

An interesting example of an antibacterial HG was synthesized from poly(*N*-isopropylacrylamide_166_-*co-n*-butyl acrylate_9_), poly(ethylene glycol), and poly(*N*-isopropyl-acryl-amide_166_-*co-n*-butyl acrylate_9_) (PEP, Figure 5A), which was additionally doped with Ag nanoparticles (PEP-AG, Figure 5B) decorated with reduced graphene oxide nanosheets [102]. The studies showed that the HG solidified at human body temperature, and after cooling, it did not return to its initial state (Figure 5).

The HG remained in its initial form—gel. The researchers also found that this lack of reversibility of thermoresponsive gelation depends on the content of Ag nanoparticles in the matrix. Briefly, a higher content of Ag nanoparticles causes a lack of reversibility. Notably, HG without the addition of Ag nanoparticles and reduced graphene oxide showed the reversibility of gelation, typical of most HGs. Additionally, the presence of Ag nanoparticles in the HG gave antibacterial properties to the material, which was targeted towards methicillin-resistant *Staphylococcus aureus* (MRSA). Owing to the liquid phase of the sol below the LCST, it is possible to administer this HG in spray form, which allows a uniform, quick and painless treatment of an infected wound, especially in extensive wounds. It has been demonstrated that a MRSA-infected wound treated with HG doped with Ag nanoparticles could be completely healed within two weeks of application.

### 3.3. Photoresponsiveness

In recent years, photoresponsive HGs have been of increasing interest in medical science. Undoubtedly, their great advantage is the noninvasive way in which light serves as a stimulating factor [103]. Additionally, by selecting adequate parameters of light radiation, for example, intensity, angle, wavelength, or exposure time, it is possible to control the physical and chemical properties of HGs [104]. In general, the photoresponsive properties of HGs can be attributed to photoresponsive moieties in polymer chains, called chromophores. Chromophores enable the optical signal that interacts with the HG to be converted into a chemical signal, and this process is called a photoreaction. The most popular photoreactions, the course of which are initiated by light, include isomerization, rearrangement, dimerization, and chain cleavage reactions [105].

The most frequently observed phenomenon is the melting of the HG structure (i.e., the gel–sol transition), caused by the applied light radiation [106]. This effect can be successfully used for more effective and localized delivery of active substances, for example, for the formation of HGs. Peptides were used for light-controlled insulin delivery [107]. UV radiation at a wavelength of 365 nm was used to crack the bonds in the HG matrix, causing the flow of the HG and the release of the active substance. Insulin release from the matrix depends on the time of exposure to UV irradiation when the exposure time is increased, and the more active substance is released linearly.

**Figure 6:**
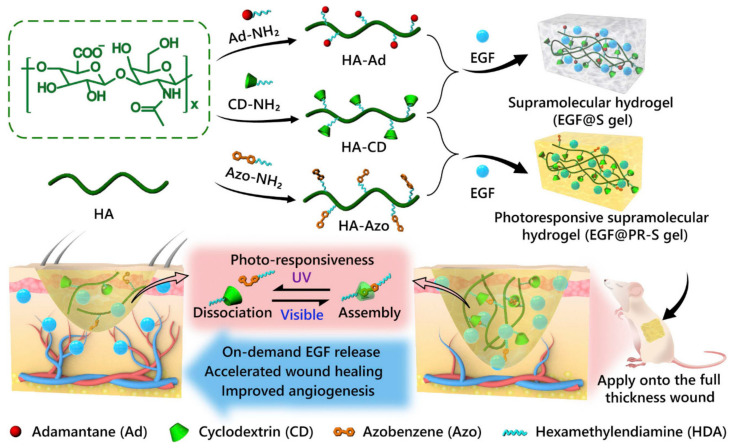
Schematic illustration of photoresponsive HG with host–guest interactions. Reprinted with permission from [108].

Photoresponsive HGs are the subject of interest for researchers regarding their therapeutic application in regenerative medicine [108]. Azobenzene was used as a photosensitive agent that was responsible for the phase transition of the HG to liquid form when exposed to UV irradiation (Figure 6). Photoisomerization of azobenzene from the *trans* to the *cis* form occurred, and in the presence of cyclodextrin and endothelial growth factor (EGF), the polysaccharide HG was formed. Studies have shown that this HG, when exposed to UV radiation, was able to accelerate the healing of wounds, allowing its application to infected wounds. Interestingly, the EGF release process was determined by the exposure time of the UV radiation, and the HG itself, after cessation of the UV radiation and exposure to visible light, was transformed from a liquid to a gel phase, making the HG longer and more easily sustained on the affected wound. Considering the possible adverse effect of UV radiation, scientists conclude that the radiation doses are too small for this to occur.

Chitosan with the addition of the antibiotic ciprofloxacin, associated with tungsten disulfide used as a photosensitizer, formed a photoresponsive HG under IR irradiation with a length of 808 nm, and the release of the active substance was achieved [109]. Furthermore, when the temperature of the surrounding cells increased, the effectiveness of antibacterial therapy was observed. As a result of this complex action, the HG can be used in the treatment of infected wounds, resulting in faster healing and recovery.

An HG that exhibits an increase in the temperature of its own and surrounding tissues was synthesized using hyaluronic and gallic acids doped with Fe^3+^ ions [110]. In this case, the photoresponsive properties of HG were used to cause the ablation of cancer cells. It has been shown that the repeated process of near-infrared irradiation of the HG results in a significant reduction of tumor cell growth. Scientists also suggest that this type of HG can be used for noninvasive treatment of topical skin cancers.

### 3.4. Conductive HGs

An important form of biomaterials for medical applications, especially for tissue engineering, is conductive HGs. Combining the ability to be biomimetic and achieve electrically conductive properties, these HGs are an interesting option for many scientists. The crucial factors here are the high water content, appropriate rheological properties such as viscosity and swelling ability, good biocompatibility, and close imitation of the physiological environment between cells in electrically active tissues [111]. The most widely used additives with conductive properties are poly(3,4-ethylenedioxythiophene) (PEDOT) [112,113,114]; polyaniline (PANI) [115,116,117]; polypyrrole (Ppy) [118,119,120]; carbon materials such as incorporated graphene [87,121] or carbon nanotubes [122]; and metal nanoparticles, including gold, silver, platinum, iron oxide, and zinc oxide [122,123]. Conductive HGs may be obtained by (i) physical crosslinking, (ii) the physical mixing of conductive material and HG, (iii) covalent crosslinking, and (iv) supramolecular crosslinking, which uses the mechanisms described for self-healing [124].

Owing to their unique abilities, conductive HGs are becoming increasingly attractive in medical science, especially in regenerative medicine and tissue engineering. The most important applications include their use as biosensors [125,126], drug carriers [87,127], and as a material simulating living tissue [128,129].

Qu et al. prepared a conductive HG containing an aniline trimer that was used as a conducting matrix for dextran delivery [130]. The mechanism of active substance release was based on the oxidative-reductive activity of the polymeric chain. According to the researchers, applying a negative electrical voltage to the matrix reduces the positively charged aniline trimer, which results in the release of negatively charged active substances, in this case, dexamethasone or indomethacin. Additionally, a decrease in HG volume was observed, which also promoted the release of drugs from the matrix. Interestingly, the observed effect could be induced by the application of voltage, and it was inhibited by the absence of applied voltage, which is described as an ‘on-off’ release mechanism. A significantly higher release of active substances at applied voltage has been observed, allowing a mechanism of on-demand release to be achieved, which can improve the effectiveness of and compliance with therapy. Analogous studies were performed using the aniline pentamer and chitosan [127]. As a result, an increase in the chitosan conductivity was observed. Additionally, the release of the active substance (dexamethasone) on demand, the ability of the polysaccharide HG to promote effective proliferation and differentiation of olfactory ecto-mesenchymal stem cells into dopaminergic neuron-like cells have been proven, attributes that can be successfully used to create conductive HGs for the treatment of nervous system disorders, such as Parkinson’s disease.

Considering the beneficial properties of HGs to simulate and reconstruct the conditions prevailing in living human tissues, tissue engineering is an important field of research. Guo et al. developed a conductive HG that uses the aniline tetramer as the factor responsible for the charge conductivity in physiological tissue [131]. As a result, their dextran- and chitosan-based HG could be effectively used as an environment for the proliferation and delivery of C2C12 into muscle tissues. The process of cell delivery to the target site occurred linearly. Thus, through its self-healing ability, HG can be successfully utilized as a supporting material for the regeneration of muscle tissues.

Xu et al. also focused on the use of HGs for cell proliferation and delivery [132]. Carboxymethyl chitosan was mixed with PEDOT, which resulted in close imitation of electrical and physicochemical conditions of nerve tissue. Afterwards, the obtained HG was loaded with neuron-like rat phaeochromocytoma cells, which were used to determine the HG’s ability to create conditions for viability and differentiation of cells. Adhesion, viability, and proliferation of the cells in the HG were successfully demonstrated, even without electrical stimulation. These studies represent a promising starting point from which to create materials for nerve tissue regeneration.

**Figure 7:**
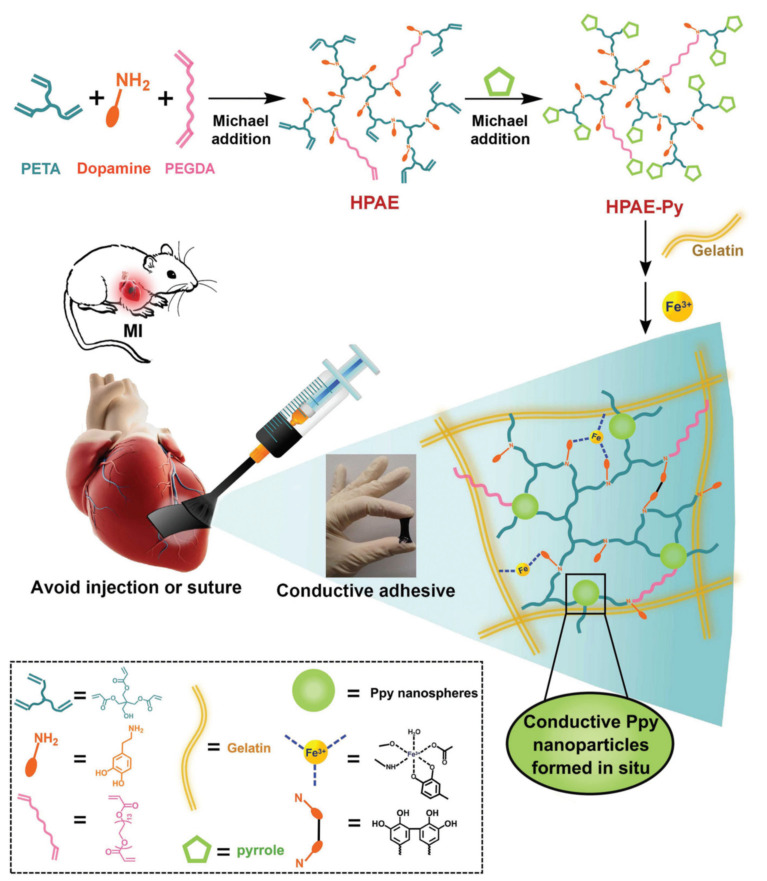
Schematic preparation and application of paintable, conductive HGs. Reprinted with permission from [133].

A fascinating paper was presented by Liang et al. [133]. The researchers prepared a special type of HG using a gelatinous matrix with Ppy particles and polymerized dopamine doped with Fe^3+^. The HG showed the ability to adhere to moist human tissues and exhibited good rheological properties (Figure 7). As a result, an adhesive conductive HG was obtained with the potential to be applied to tissues by brush painting. The material was used to carry out conductivity tests on live and beating heart muscle tissue in the state of infarction. The results suggested that the obtained HG may support the electrophysiological conductivity of cardiac muscle tissue. It was also suitable for revascularization of myocardial muscle during myocardial infarction. This is an important step in research on tissue engineering of the myocardium.

### 3.5. 3D Bioprinting

Advances in technology enable the ongoing improvement of many fields of science, including medicine and pharmacy. The idea of a 3D printer has not only resulted in greater availability of biomaterials, since such printers are widely available, but also in more personalized printed biomaterials. In addition, 3D printing has a better print resolution, which results in a more accurate and detailed design and obtaining of HG material [134]. It is worth mentioning, that 3D bioprinting is a very good method for bioprinting HGs scaffolds containing living cells [135,136]. It is not surprising that 3D bioprinting has been successfully adapted for use in tissue engineering and regenerative medicine.

It is worth distinguishing how 3D printing differs from 3D bioprinting. Both of these processes enable the creation of objects on the system ‘layer by layer’. The main difference depends on the type of printing material used, which constitutes the so-called ‘ink’. In the case of regular 3D printing, classic materials such as polymers (polyvinyl alcohol, polylactic acid, nylon) or other composites are used, while in 3D bioprinting, bioinks charged with cells are used [137]. Due to the unique properties of HGs, mainly their ability to incorporate cells and their high viability, HGs have found broad application in bioink production. The most widely used HGs as bioinks include alginates [138,139], gelatine methacrylate [140,141,142], cellulose [143,144], and chitosan [145,146]. Additionally, with bioprinting, live tissue can be incorporated into the system, which could not be achieved by standard 3D printing techniques.

Undoubtedly, one of the most important elements of bioprinting is the proper choice of bioink. Several requirements must be met for bioprinting and are crucial for creating the appropriate tissue model. These requirements include first and foremost biocompatibility, biodegradation and the lack of cytotoxicity. These factors are important because the HG should not be harmful to the surrounding tissues or to the cells that are incorporated in it. For printed tissue to be able to reproduce the functions of natural tissue as faithfully as possible, it is also important to select the appropriate pore size in the HG matrix so that the cells have room to multiply and to provide space for the transfer of nutrients and growth factors. Additionally, an essential feature of the HG is its appropriate rheological properties, which should be included in the category of pseudoplastic shear-thinning fluids. The viscosity and surface tension parameters are also crucial in this aspect. All these features affect the so-called ‘printability’ of the HG, which is especially important for the selection of the bioprinting technique. An HG that can be recognized as a good bioink should be in liquid form before printing and should form gel only when triggered. This property facilitates the dispersion of the bioink and increases the printing resolution.

**Figure 8:**
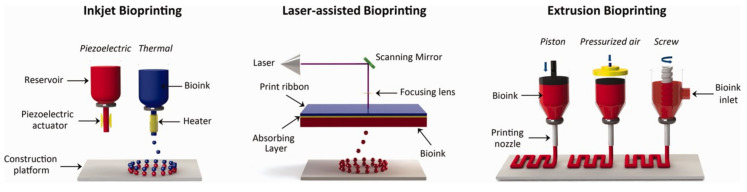
Examples of 3D bioprinting methods. Reprinted with permission from [148].

Over the years, several methods have been developed for tissue bioprinting. The four most important methods are (i) inkjet bioprinting, (ii) extrusion bioprinting, (iii) laser-assisted bioprinting, and (iv) stereolithography bioprinting (Figure 8) [147].

Inkjet bioprinting is based on the same principle as traditional inkjet printers. A bioink cartridge, a cell-laden HG with cell growth factors, is inserted into the device (Figure 8). Considering the way the bioink comes out of the nozzle, continuous inkjet (CIJ) and drop-on-demand (DOD) methods can be distinguished. In the first method, the HG comes out of the dosing device in a continuous stream; in the second method, the droplets come out by triggering, which makes the process easier to control. In addition, the DOD can be divided according to the triggering factor: thermal [149,150], electrostatic [151], or piezoelectric [108,152]. These factors are usually applied to the cartridge outlet. The thermal agent heats a drop of ink, and it creates a bubble of gas, which breaks and pushes the drop of bioink onto the printed surface, while the vacuum created in the nozzle sucks in another portion of toner. In electrostatic bioprinting, there is a special plate in the nozzle, which deforms after applying sufficient voltage between it and the electrode, causing a change in the nozzle capacity and pushing out the bioink drop. Piezoelectric printing uses an inverse piezoelectric phenomenon, in which the size of a crystal with these properties changes under the influence of the applied voltage. As a result, as in previous methods, a drop of bioink is created, and it is printed on the surface. Clear advantages of this kind of bioprinting are the low cost of the process, high resolution, and good cell viability.

The principle of extrusion bioprinting is based on the syringe mechanism (Figure 8). The bioink cell-laden HG placed in the reservoir is pressed through the nozzle by the pressure generated by the piston [153,154]. Due to its uncomplicated process, this method has an undoubted advantage over the others. It allows the bioprinting of human tissues in their original size as well as the use of more viscous solutions of bioinks. However, this results in a relatively low print resolution.

Laser-assisted bioprinting uses a laser beam to create spatial biostructures (Figure 8). It utilizes the unique properties of laser radiation, such as coherence and monochromaticity. This provides the possibility of precise and accurate reproduction of the desired model object in reality. There are several laser printing systems, of which the most widely used are absorbing film-assisted laser-induced forward transfer (AFA-LIFT) [155] and matrix-assisted pulsed laser evaporation direct writing (MAPLE-DW) [156]. Despite the multitude of methods, the general principle and components used are similar. When the laser beam impacts the print ribbon, the energy is absorbed, and high gas pressure is created, which results in the generation and detachment of the bioink drop and its placement on the print surface. The difference between these two methods is particularly in the energy of the laser beam. In the AFA-LIFT method, a stronger laser beam is used, while in the MAPLE-DW method, a weaker beam does not penetrate deeply into the bioink. In addition, the absorbing layer in the print ribbon in the AFA-LIFT method is doped with metals and, in the second layer (MAPLE-DW), with biopolymers.

Stereolithography bioprinting is based on a gradual, layer-by-layer formation of a programmed structure using radiation, usually in the UV range or laser beam [157,158]. This is achieved through the precise light beam application on the surface of the bioink layer, which is subsequently crosslinked and solidified, after which it is lowered accurately to the height of the created layer and covered with a fresh and uncross-linked bioink, from which another layer can be created until the intended object is produced.

The whole process of producing new tissue consists of three main stages: preprocessing, proper bioprinting, and postprocessing. Preprocessing includes all the steps of acquiring as much information as possible about the reconstructed or de novo created tissue or organ. A variety of computer imaging methods, magnetic resonance, and optical microscopy are used in this stage. On the basis of the gathered information, it is possible to successfully create a reliable computer model of the tissue to be physically created by a 3D bioprinting process. The stage of postprocessing is also an important step in creating new biological material. It depends on maturing the produced tissue to achieve the full ability to replace a living organ [159]. Apart from creating models of various tissues for medical use, models for testing drugs in laboratories [160,161] and wound dressings have been developed [162,163].

## 4. Conclusions

Interest in HGs is not diminishing; on the contrary, it is increasing from year to year, which is easily observed in search engines for scientific papers. It is also encouraging that, due to their unique properties, HGs have found irreplaceable use in regenerative medicine and tissue engineering. With rapid technological progress and the discovery of newer fields of science, especially medical fields, it seems clear that the HG applications known today are only the tip of the iceberg. This potential is further amplified by the fact that each HG structure can be modified, which gives an infinite number of combinations and unlimited possibilities for their use in medical science.

## Figures and Tables

**Table 1:** Methods of porous HGs preparation

Method	Mechanism	Advantages	Limitations
Porogen templating	The usage of porogens during the gelation process, that are removed after HGs formation.	Simple process; good pore size control	Difficulties with porogen removal
Gas foaming	High-speed stirring of gel, which generates bubbles, or addition of substance, that produces gas particles through a chemical reaction.	Simple process; inexpensive method	Insufficient possibility of pore size control
Bicontinuous emulsion templating	Preparation of emulsion, where the aqueous phase is a mixture of monomers for polymerization.	Simple process	Various pores size; water soluble polymers
Cryogelation	Polymerization at very low temperatures (formation of crystals). During controlled heating, crystals are melt and pores are formed.	More interconnective porogen structure	Use of sub-zero temperatures
3D printing	3D printing of HG matrix with specially planned and strictly defined pore sizes.	Controllable pores size	Insufficient resolution of 3D printers
Electrospinning	Usage of electric charge to obtain porous structure of polymeric HG.	Microscale and macroscale process	Relatively slow process
Freeze-drying	Preparation of an oil-in-water emulsion from which water phase is removed during freeze-drying (formation of pores).	Good pore size control	Water insoluble polymers
Inverse opal hydrogelation	Preparation of a 3D pattern from colloidal particles, between which polymer solution is poured in, followed by removal of the template after polymerization	Interconnected pores	Selection of colloidal particles

**Table 2:** Classification of self-healing mechanisms

Type of Self-Healing Mechanism		
Intrinsic		Extrinsic
Physical (Non-Covalent)	Chemical (Covalent)	
Electrostatic interactions [66,67]	Acylhydrazone bonds [68,69]	Microcapsule [70,71]
Host–guest interactions [72,73,74]	Boronate-diol complexation [75,76]	
Hydrogen bond [77,78,79]	Diels–Alder click chemistry [80,81,82]	
Hydrophobic interactions [83,84]	Disulfide bonds [85,86]	
π–π stacking [87,88]	Imine bonds (Schiff base) [89,90]	
